# Assessment of professional competencies of Peruvian physicians: A scoping review of published studies

**DOI:** 10.1371/journal.pone.0299465

**Published:** 2024-05-23

**Authors:** Cristhian Rojas-Miliano, Shalom Aperrigue-Lira, Fernanda Barriga-Chambi, Edson Huamani-Merma, Alvaro Taype-Rondan

**Affiliations:** 1 Sociedad Científica de Estudiantes de Medicina del Centro, Universidad Nacional del Centro del Perú, Huancayo, Peru; 2 Sociedad Científica de Estudiantes de Medicina Agustinos, SOCIEMA; Universidad Nacional de San Agustín de Arequipa, Arequipa, Peru; 3 Asociación Científica de Estudiantes de Medicina Humana del Cusco, ASOCIEMH CUSCO, Universidad Nacional de San Antonio Abad del Cusco, Cusco, Peru; 4 Unidad de Investigación para la Generación y Síntesis de Evidencias en Salud, Universidad San Ignacio de Loyola, Lima, Peru; 5 EviSalud–Evidencias en Salud, Lima, Peru; Norbert Wiener University, PERU

## Abstract

**Introduction:**

Evaluating the professional competencies of Peruvian doctors is crucial for proposing necessary improvements. However, there is a lack of knowledge regarding the specific characteristics and competencies that are assessed in these studies.

**Objective:**

The objective of this study is to characterize published studies focusing on the assessment of professional competencies among physicians in Peru.

**Methods:**

A comprehensive scoping review was conducted, encompassing scientific journal publications that evaluated the professional competencies of physicians in Peru. The search was performed in PubMed, Google Scholar, Scopus, and SciELO, with the review period extending until 2022. The identified competencies were classified using the Accreditation Council for Graduate Medical Education (ACGME) and the Ministry of Health of Peru (MINSA) frameworks. The findings were presented using absolute and relative frequency measures.

**Results:**

A total of forty-nine studies focused on the assessment of professional competencies among physicians were identified, indicating an upward trend over the years. The primary focus of these studies was on evaluating competencies related to medical knowledge (79.6% according to ACGME classification) and the treatment of health problems (57.1% according to MINSA classification). However, there was a noticeable lack of emphasis on assessing behavioral competencies such as ethics, professionalism, and communication. Most of the included studies (65.3%) were exclusively conducted in Lima. Among the studies that disclosed their funding sources, 61% were self-funded.

**Conclusion:**

Most studies primarily concentrated on evaluating knowledge-based competencies, specifically in the areas of diagnosis and treatment. There is a scarcity of studies assessing other important competencies. Additionally, centralization and limited funding appear to be areas that require improvement in the evaluation of professional competencies among Peruvian physicians.

## Introduction

The medical profession bears significant responsibility for the health attention of the population [1]. To ensure a proficient practice, physicians must acquire the necessary competencies to utilize their knowledge, skills, attitudes, and judgment for effective interventions [2]. Nonetheless, inadequacies have been detected in the medical skills acquisition across numerous countries, Peru included, where shortcomings in areas like knowledge, behavioral aptitudes, research proficiencies, and others have been documented [3–8].

Previous literature reviews have examined the existing research on medical competencies in specific countries. For instance, in the United Kingdom, limited studies have focused on evaluating the interactional and interpersonal aspects, systemic and technological components, and personal training of physicians [9]. Similarly, a systematic review investigated the medical competencies outlined by the Accreditation Council for Graduate Medical Education (ACGME) for medical residents, revealing that patient care and medical knowledge were the most frequently assessed areas [10]. However, there is currently a dearth of reviews that have evaluated Peruvian studies on medical competencies.

Identifying and describing the published studies on professional competencies among physicians and final-year medical students in Peru is crucial for recognizing knowledge gaps and systematizing the existing information. It will provide guidance for the implementation of national strategies aimed at enhancing doctors’ training with the necessary professional skills. With this objective in mind, the present study aims to conduct a comprehensive scoping review that characterizes the published studies on professional competencies in physicians and final-year medical students in Peru.

## Methods

### Design and eligibility criteria

A scoping review was carried out to evaluate scientific articles reporting original findings (original studies, editorials, or other article types) published in scientific journals until 2022. The review centered on studies that examined the professional competencies of physicians (including general practitioners, residents, and specialists) from Peru. We also included final-year medical students (interns) which are a few weeks away from finishing medical school, as their competencies were expected to be relatively consistent with those of licensed physicians. Language restrictions did not apply.

Intervention studies lacking a baseline measurement of competencies before implementation were excluded. Further, studies that focused on a broader population (such as any-year-medical students or healthcare professionals in general) that did not present data on competencies assessed separately from the target populations (interns or physicians) were also excluded.

### Operational definition of competencies

A study was considered to have assessed professional competencies in the field of medicine if it addressed any of the elements outlined by the Accreditation Council for Graduate Medical Education (ACGME) [11] or the Ministry of Health of Peru (MINSA, by its acronym in Spanish) [12].

On the one hand, the ACGME [13] provides a practical and comprehensive classification of competencies (including hard and soft skills) that’s a reference for the development of postgraduate medical education programs (medical residency) [14]. This classification was proposed in collaboration with the American Board of Medical Specialties and other organizations interested in postgraduate medical education in the United States [15]. As a result, the ACGME proposes 25 items [16] divided into six core competencies: 1) patient care, 2) medical knowledge, 3) practice-based learning and improvement, 4) system based-learning, 5) professionalism, and 6) communication and interpersonal skills [11].

Similarly, in Peru, MINSA issued a ministerial resolution in 2020 outlining a set of competencies expected of general practitioners to ensure high-quality higher education provision. The competency framework was developed by analyzing the population’s health needs and the healthcare system characteristics, among other related considerations. Subsequently, these competencies were established through a collaborative process involving educational institutions, healthcare providers, and civil society.

This classification is divided into 13 competencies: 1) Carry out the clinical evaluation and establish a work plan, 2) Carry out the comprehensive treatment of low/high-complexity health problems, 3) Carry out actions for the best recovery of the person with sequelae of physical, mental or social damage, 4) Promote changes in individual, collective, and surroundings; 5) Carry out health interventions to reduce exposure, risks, and damages that affect individual health, and public health; 6) Exercise their profession according to the Peruvian health system, 7) Participate in the training of students and the strengthening of capacities of human resources in health, 8) Generate new knowledge, which contributes to the solution of health problems and decision-making, 9) Apply technology and innovation scientifically founded, 10) Demonstrate commitment to well-being and health, 11) Establish professional relationships with the person, family, and community, 12) Influence and motivate people with respect and equity, 13) Establish cooperative relationships, sharing knowledge and resources. Furthermore, MINSA further categorizes these competencies into technical skills (1 to 9) and behavioral skills (10 to 13). The detailed operational definitions for each competency can be found in **S1 Table.**

### Literature search

The search was conducted from February 5 to March 25, 2023, utilizing PubMed, Scopus, SciELO, and Google Scholar. Detailed search strategies are in **S2 Table**.

### Selection of studies

The articles identified through the search strategies were imported into Rayyan Software, where duplicates were eliminated. Subsequently, three authors (FBC, SAL, EHM) independently evaluated the articles based on their titles and abstracts to identify potentially eligible ones. These selected articles were then thoroughly reviewed in full text, and those that fulfilled the inclusion criteria were chosen. Discrepancies were resolved through consultation with a fourth author (CRM).

### Data extraction

The data of interest in the included articles were extracted to a Microsoft Excel sheet in duplicate by fourth authors (FBC, SAL, EHM, CRM). Discrepancies were resolved by another author (ATR).

The following variables were extracted from the studies: author, publication year, article type (original, letter to the editor, other), study design (cross-sectional, descriptive or analytical, longitudinal, intervention), location (city of the study conduction), the sample size of the target population, included population (internists, general practitioners, residents, and specialists), study context (university, health center or hospital, virtual survey, events such as courses, congresses, workshops, information from the National Medical Examination (ENAM, by its acronym in Spanish), journal of publication (Peruvian, foreign), journal indexing in Scopus (yes, no), funding (yes, no), the language of publication (Spanish and English), evaluated competence, the method used to assess the competence and the type of competence evaluated according to the ACGME and MINSA classification. The complete database is located in **S3 Table**.

### Ethics statement

Since this is a scoping review of previously published summary data, ethical approval for this study was not needed.

### Summary of results

For the analysis of the results, narrative descriptions were used, as well as absolute and relative frequency measures. Analyzes were performed using the Microsoft Excel program.

## Results

**Fig 1:**
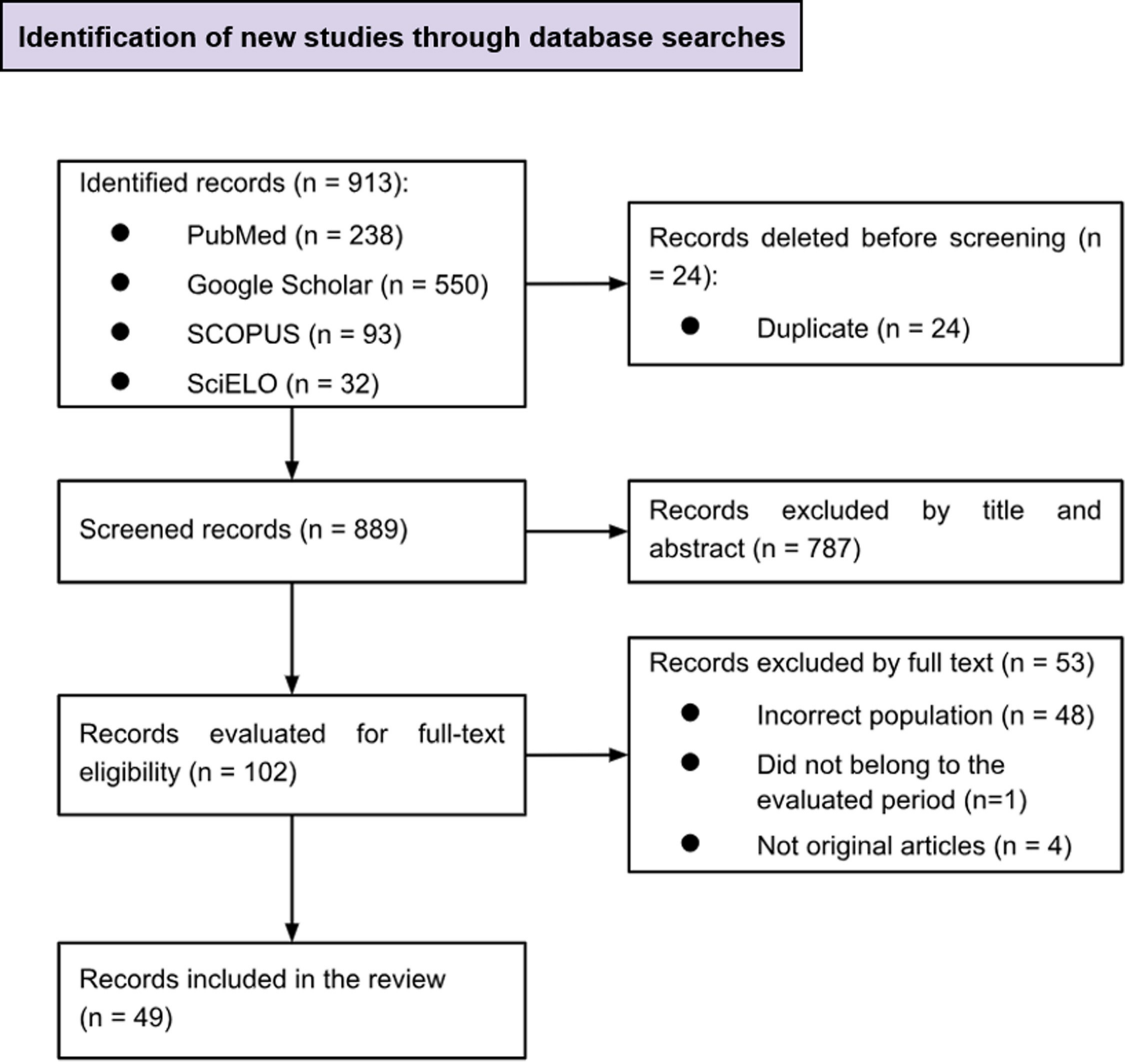
Flowchart of the article selection process.

A total of 913 records were identified, of which 49 studies that met the selection criteria were finally included **(Fig 1)**.

Of the included studies, 26 (53.0%) employed a descriptive cross-sectional design, while 19 (38.8%) utilized an analytical cross-sectional approach. In geographical distribution, 32 studies (65.3%) were conducted exclusively in Lima, the capital city of Peru. Furthermore, the sample sizes varied widely, ranging from 14 to 30,750 participants, with a median of 113.

Concerning the assessment tools employed to evaluate competencies, 22 studies (44.9%) utilized surveys developed by the authors. Among these, ten studies did not specify the source of instrument creation; five articles indicated that surveys were previous-research based; and seven were developed according to specific criteria, such as technical standards or official documents. Additionally, seven studies (14.3%) utilized ENAM data. In 42 studies (85.7%), competencies were assessed directly, while in seven studies, the evaluation focused on self-perception or perception by others regarding competence. Detailed characteristics of each study are in **Table 1** includes the corresponding references for each study.

**Table 1:** Characteristics of the included studies (n = 49 studies).

Author (year)	Study design	City of the study	N	Evaluated competence	Competency assessment method
**Last-year medical students (interns) about to finish medical school:**					
Aparicio (2011) [17]	Intervention	Lima	72	Knowledge of childbirth, partogram and application in the preparation of the partograph	Survey prepared by the authors
Arenas-Significación (2014) [18]	Analytical cross-sectional	Lima	146	Knowledge of general medicine	ENAM
Cieza-Zeballos (2010) [19]	Analytical cross-sectional	Peru	Interns at 23 medical schools	Knowledge of general medicine	ENAM
Flores-Cohaila J (2022) [20]	Analytical cross-sectional	Peru	46	Knowledge of general medicine	ENAM
Huamaní C (2011) [21]	Analytical cross-sectional	Peru	6556	Knowledge of general medicine	ENAM
Málaga (2020) [22]	Descriptive cross-sectional	Lima	63	Medical empathy	Jefferson Medical Empathy Scale for Medical Students
Mendoza Chuctaya (2018) [23]	Descriptive cross-sectional	Cusco	377	Knowledge of general medicine	ENAM
Mendoza Chuctaya (2019) [24]	Analytical cross-sectional	Peru	21296	Knowledge of general medicine	ENAM
Mendoza-Chuctaya (2021) [25]	Analytical cross-sectional	Peru	30750	Knowledge of general medicine	ENAM
Prialé (2022) [26]	Descriptive cross-sectional	Lima	44	Physician-patient relationship, diagnosis, differential diagnoses, work plan, therapeutic measures, anamnesis, interview and physical examination	Multimodal clinical simulation of four specialties (Internal Medicine, General Surgery, Gynecology and Pediatrics) [escenario clínico]
Tarazona-Pedreros (2021) [27]	Analytical cross-sectional	Lima	71	Knowledge in palliative care	Palliative Care Knowledge Test (PCKT) Survey
Vera-Ponce (2022) [28]	Analytical cross-sectional	Unspecified	45	Statistics knowledge	Survey "Assessment Resource Tools for Improving Statistical Thinking" (ARTIST)
**General practitioners:**					
Acuña Ortiz (2016) [29]	Descriptive cross-sectional	Lambayeque	145	Knowledge in fibromyalgia (diagnosis and treatment)	Survey prepared by the authors based on the criteria for diagnosis and treatment for fibromyalgia of the American College of Rheumatology (ACR)
Bustamante-García (2021) [30]	Analytical cross-sectional	Unspecified	289	Self-perception of managerial competencies	Questionnaire “How Dutch medical specialists perceive the competencies and training needs of medical residents in healthcare management”
Chiang (2015) [31]	Descriptive cross-sectional	Lima	103	Knowledge about childhood tuberculosis (transmission, clinical presentation, diagnosis, prevention and treatment)	Survey prepared by the authors based on the "Technical Health Standard for the Control of Tuberculosis" prepared by the Ministry of Health of Peru
Correa-Carhuachin (2011) [32]	Descriptive cross-sectional	Lima	15	Knowledge about non-alcoholic fatty liver	Survey prepared by the authors
Correa-Carhuachin (2014) [33]	Descriptive cross-sectional	Lambayeque	60	Knowledge of non-alcoholic fatty liver (diagnosis, treatment, prognosis, etc.)	Survey prepared by the authors
Nieto-Gutierrez (2020) [34]	Descriptive cross-sectional	Lima	425	Self-perception of competencies for clinical practice in general medicine (outpatient consultation, immediate medical emergency care, performing practical procedures, applying evidence-based medicine)	Survey prepared by the authors based on the basic competencies for Medicine established by the Tuning Project adapted for Latin America
Taype-Rondán (2015)* [35]	Descriptive cross-sectional	Lima	268	Self-perception of clinical skills (perform a physical examination, write a medical history, etc.)	Survey prepared by the authors
Taype-Rondán (2016) [36]	Analytical cross-sectional	Lima	117	Self-perception of training in performing medical procedures (clinical, surgical and gynecological-obstetric)	Survey prepared by the authors based on the medical competencies recommended by the Commission for the Accreditation of Faculties or Schools of Human Medicine in Peru, Scottish Deans’ Medical Education Group, Tomorrow’s Doctors, Association of American Medical Colleges, the Tuning project and CanMEDS
Zafra-Tanaka (2018) [37]	Analytical cross-sectional	Lima	432	Self-perception of competencies to perform diagnostic tests for cervical cancer	Survey prepared by the authors
Zafra-Tanaka (2019) [38]	Analytical cross-sectional	Lima	434	Self-perception of competences in the diagnosis and treatment of mental health disorders	Survey prepared by the authors according to the guidelines of the World Health Organization (WHO).
Zavala (2012) [39]	Descriptive cross-sectional	Lima	1389	Knowledge about patient autonomy and the right to refuse treatment	Survey prepared by the authors based on previous studies
**Residents**					
Agreda-Carrillo (2017) [40]	Descriptive cross-sectional	Lima	113	Knowledge and practices about major depression	Questionnaire validated in a previous study and questions prepared by the authors based on previous studies
Mayo (2019) [41]	Descriptive cross-sectional	Lima	100	Medical empathy	Jefferson Empathy Scale for Health Professionals
Olascoaga (2019) [42]	Longitudinal	Lima	14	Clinical skills (history, physical examination, professionalism, organization, clinical judgment, communication skills)	Mini Clinical Examination (Mini-CEX) [clinical scenario]
Riveros-Ruiz (2020) [43]	Descriptive cross-sectional	Lima	271	Perception and self-perception of their role in teaching residents	Questionnaire "resident as educator"
Ticse (2019) [44]	Intervention	Lima	25	Communication skills	Structured objective clinical examination (OSCE) of communication based on ICCAT (connect, identify, understand, agree, help). [clinical scenario]
Yagui (2021) [45]	Analytical cross-sectional	Lima	171	Knowledge and practices on prevention of infections associated with health care	Survey prepared by the authors based on previous studies
**Specialists:**					
Cuba-Fuentes (2016) [46]	Descriptive cross-sectional	Lima	40	Knowledge and perceptions about quaternary prevention.	Survey prepared by the authors
Olascoaga (2021) [47]	Analytical cross-sectional	Lima	70	Teaching performance in residence	MEDUC-PG14 Questionnaire
**Physicians (unspecified)**					
Cherrez Ojeda (2016) [48]	Descriptive cross-sectional	Lima	52	Self-perception of communication strategies and skills	Survey developed by a panel of experts from the Italian Society of Respiratory Medicine
Condor-Rojas (2020) [49]	Analytical cross-sectional	Lima	196	Hand hygiene knowledge	Survey prepared by the authors based on institutional recommendations for handwashing
Cordova (2018) [50]	Intervention	Cajamarca	20	Knowledge and practical skills in Helping Babies Breathe (HBB)	Objective Structured Clinical Questionnaires (OSCE) developed by the American Academy of Pediatrics [clinical scenario]
Ochoa-Alencastre (2009) [51]	Descriptive cross-sectional	Peru	121	Knowledge about educational methodology for the training of adults of health personnel	Survey prepared by the authors
Pace (2006) [52]	Descriptive cross-sectional	Unspecified	35	Knowledge of abortion law and practice	Survey used in previous study
Paredes (1996) [53]	Descriptive cross-sectional	Lima	40	Medical prescription in cases of acute childhood diarrhea	Survey prepared by the authors
Romani Romani (2016) [54]	Descriptive cross-sectional	Tacna	62	Knowledge, perceptions and practices on the detection of respiratory symptoms	Survey prepared by the authors based on the "Technical Health Standard for Comprehensive Care of People Affected by Tuberculosis"
Snowden (1988) [55]	Descriptive cross-sectional	Lima	105	Knowledge and perceptions about methods of periodic reproductive abstinence	Survey prepared by the authors
Others (more than one population)**					
Canelo (2007): R & S [56]	Analytical cross-sectional	Lima	62	Knowledge and practices of evidence-based medicine	Survey prepared by the authors based on previous studies
Castro-Suarez (2022): R & S [57]	Descriptive cross-sectional	Unspecified	145	Knowledge about the treatment of the behavioral variant of frontotemporal dementia	Survey prepared by the authors
Cherrez Ojeda (2013): R & S + [58]	Analytical cross-sectional	Unspecified	93	Knowledge about obstructive sleep apnea	OSAKA questionnaire
Custodio (2018): GP & S [59]	Descriptive cross-sectional	Lima	201	Knowledge of frontotemporal dementia	Adapted survey developed by Gleichgerrcht et al.
Flores (2022): GP & S [60]	Analytical cross-sectional	Lima	382	Knowledge about rare diseases	Survey prepared by the authors based on previous studies
Quispe (2019): R & S [61]	Descriptive cross-sectional	Lima	30	Medical communicative competence during the clinical interview	Communication Assessment Tool
Montes Teves (2002): GP & S [62]	Analytical cross-sectional	Lima	152	Knowledge of Helicobacter pylori infection	Survey prepared by the authors
Tejada-Hidalgo (2018): R & S [63]	Descriptive cross-sectional	Callao	90	Knowledge of obstructive sleep apnea	OSAKA questionnaire
Vásquez-Silva (2015): R & S [64]	Descriptive cross-sectional	Lima	211	Mastery, access, use of information and communication technologies	Modified survey used in a previous study
Zea-Vera (2012): I & R [65]	Descriptive cross-sectional	Lima	172	Knowledge of epidemiological risk indicators	Survey prepared by the authors based on previous studies

*Letter to the editor.

** **R**: Residents; **S**: Specialists; **GP**: General practitioners; **I**: Interns and residents.

**N**: Number of participants.

ENAM: National Medicine Exam.

Among the studies included in the analysis, 14 (28.6%) were focused on general practitioners, while 13 (26.5%) were conducted among residents. Furthermore, a significant proportion of the studies, accounting for 26 (53.0%), were published in international journals, indicating a wide dissemination of the findings. Additionally, 35 (71.4%) of the studies were published in journals indexed in Scopus, reflecting their recognition and scholarly impact. It is worth mentioning that financial support was reported in 15 studies (30.6%), indicating external funding sources contributing to the research endeavors (**Table 2**)

**Table 2:** Grouped characteristics of the included studies (n = 49 studies).

Characteristics	n (%)
Population*	
Last-year medical students (interns) about to finish medical school	13 (26.5)
General practitioners	14 (28.6)
Resident physicians	13 (26.5)
Specialist physicians	11 (22.4)
Physicians (unspecified)	8 (16.3)
**Context in which the study was conducted**	
University	6 (12.2)
Health centers/hospital	18 (36.7)
Medical meetings (congresses, courses, workshops)	10 (20.4)
Online survey	5 (10.2)
Information from the National Medicine Exam (ENAM)	7 (14.3)
Not specified	3 (6.1)
**Journal in which the study was published**	
Peruvian	24 (49.0)
Foreign	25 (51.0)
**The journal is indexed in Scopus**	
No	14 (28.6)
Yes	35 (71.4)
**Article language**	
Only Spanish	30 (61.2)
Only English	15 (30.6)
Spanish and English	4 (8.2)
**Funding**	
Received financing	15 (30.6)
Self-funded	23 (46.9)
Not specified	11 (22.4)
**Year of publication**	
Before 2003	3 (6.1)
2003–2007	2 (4.1)
2008–2012	7 (14.3)
2013–2017	12 (24.5)
2018–2022	25 (51.0)

* Studies may include a population of physicians belonging to more than one category.

The assessment of medical competencies revealed a range of 0 to 5 competencies evaluated, following the ACGME classification. Among the included studies, a significant proportion (79.6%) focused on competencies associated with "medical knowledge." The remaining competencies were assessed by varying numbers of studies, with each competency being evaluated by between 2 and 9 studies.

Following the MINSA classification, the evaluated studies encompassed a range of 0 to 9 competencies. Notably, 28 studies (57.1%) focused on competencies associated with dimension 1, which involves clinical assessment and a work plan establishment. Similarly, 28 studies (57.1%) addressed competencies related to dimension 2, which entails the comprehensive treatment of low/high-complexity health issues. The remaining were evaluated by varying numbers of studies, ranging from zero to seven.

Technical competencies were evaluated in 41 studies (83.7%), while behavioral competencies were assessed in eight studies (16.3%). Regarding technical competencies, only one study (2.0%) evaluated dimension 8, which pertains to generating new knowledge to address health issues and aid decision-making. Additionally, just one study (2.0%) assessed dimension 9, which involves applying technology and scientifically grounded innovations. However, none of the studies evaluated dimension 12, which encompasses influencing and motivating individuals with respect and equity, by MINSA (**Table 3**).

**Table 3:** Types of competencies assessed in the studies according to the ACGME and MINSA classifications (n = 49 studies).

	ACGME						MINSA												
							Technical skills									Behavioral competencies			
**Source/competition**	1	2	3	4	5	6	1	2	3	4	5	6	7	8	9	10	11	12	13
**Last-year medical students (interns) about to finish medical school:**																			
Aparicio Ponce JR (2011) [17]		X					X												
Arenas-Significación F (2014) [18]		X					X	X											
Cieza-Zeballos JA (2010) [19]		X					X	X											
Flores-Cohaila J (2022) [20]		X					X	X											
Huamaní C (2011) [21]		X					X	X											
Málaga G. (2020) [22]				X	X														
Mendoza Chuctaya G (2018) [23]		X					X	X											
Mendoza Chuctaya G (2019) [24]		X					X	X											
Mendoza Chuctaya G (2021) [25]		X					X	X											
Prialé A (2022) [26]	X	X	X	X			X	X								X	X		
Tarazona-Pedreros DE (2021) [27]		X						X	X										
Vera-Ponce VJ (2022) [28]		X																	
**General practitioners:**																			
Acuña Ortiz FA (2016) [29]		X					X	X											
Bustamante-García M (2021) [30]						X						X							
Chiang SS (2015) [31]		X					X	X											
Correa-Carhuachin V (2011) [32]		X					X	X											
Correa-Carhuachin KV (2014) [33]		X					X	X											
Nieto-Gutierrez W (2020) [34]	X	X		X	X		X	X				X				X	X		
Taype-Rondán A (2015) [35]	X	X		X	X		X	X		X	X	X		X		X	X		X
Taype-Rondán A(2016) [36]		X					X	X											
Zafra-Tanaka JH (2018) [37]		X					X												
Zafra-Tanaka JH (2019) [38]		X					X	X											
Zavala Sarrio S (2012) [39]					X											X			
**Residents**																			
Agreda-Carrillo ER (2017) [40]		X					X	X											
Mayo GV (2019) [41]				X	X											X			
Olascoaga AC (2019) [42]		X	X	X	X	X	X	X								X	X		
Riveros-Ruiz J (2020) [43]		X											X						
Ticse-Aguirre RW (2019) [44]				X													X		
Yagui M (2021) [45]		X									X								
**Specialists**																			
Cuba-Fuentes MS (2016) [46]		X																	
Olascoaga AC (2021) [47]													X						
**Physicians (unspecified)**																			
Cherrez Ojeda I (2016) [48]	X			X				X									X		
Condor-Rojas Y (2020) [49]		X									X								
Cordova E (2018) [50]		X	X					X											
Ochoa-Alencastre M (2009) [51]	X										X								
Pace L (2006) [52]		X						X	X			X							
Paredes P (1996) [53]		X					X	X											
Romani Romani FR (2016) [54]		X					X				X								
Snowden R (1988) [55]	X	X								X	X								
**More than one population**																			
Canelo Aybar GC (2007) [56]		X																	
Castro-Suarez S (2022) [57]		X					X	X											
Cherrez Ojeda I (2013) [58]		X					X	X											
Custodio N (2018) [59]		X					X												
Flores A (2022) [60]		X					X	X											
Quispe (2019) [61]				X													X		
Montes Teves PA (2002) [62]		X					X	X			X								
Tejada-Hidalgo K (2018) [63]		X					X	X											
Vásquez-Silva L (2015) [64]															X				
Zea-Vera A (2012) [65]		X																	
**Total**	**6**	**39**	**3**	**9**	**6**	**2**	**28**	**28**	**2**	**2**	**7**	**4**	**2**	**1**	**1**	**6**	**7**	**0**	**1**

***ACGME Competencies*:** *1) Patient care*, *2) Medical knowledge*, *3) Practice-based learning and improvement*, *4) Interpersonal and communication skills*, *5) Professionalism*, *6) Systems-based learning*.

***Competences according to MINSA:*** *1) Carry out the clinical evaluation and establish a work plan; 2) Carry out comprehensive treatment*, *3) Carry out actions for the best recovery of the person with sequelae of physical*, *mental or social damage*. *4) Promote changes in individual*, *collective and surroundings*, *5) Carry out health interventions to reduce exposure*, *risks and damages*, *6) Practice their profession in accordance with the Peruvian health system*, *7) Participate in the training of students and in strengthening the capacities of human resources in health*, *8) Generate new knowledge, which contributes to the solution of health problems and decision-making*, *9) Apply technology and scientifically founded innovation*. *10) Demonstrate commitment to the well-being and health of people and society*, *11) Establish professional relationships with the person*, *family and community*, *12) Influence and motivate people with respect and equity*, *13) Establish cooperative relationships*, *Sharing knowledge and resources*.

## Discussion

The scientific output concerning medical competencies in Peru demonstrates a notable upward trend, indicating a growing interest in the field, and appears to be accompanying a growing research-publishing culture in Peru [66].

The rise in scholarly publications in this field serves as an encouraging indication that there is a growing emphasis on the assessment and enhancement of medical education in Peru. This trend holds promising prospects for enhancing the quality of healthcare services delivered to the populace. The significance of this development is in light of concerning reports revealing a remarkable failure rate among final-year medical students in the ENAM examination [67] and suboptimal levels of public satisfaction with healthcare [68].

Regarding geographical distribution, the majority of studies were predominantly conducted in Lima, the capital city of Peru, while limited representation from other regions was observed. This centralized pattern has been noted in various research domains [69] and could be attributed to resource constraints, and limited research expertise in those regions [70]. It is imperative to research medical competencies in other areas of the country to assess and enhance the quality of medical education, as well as propose interventions aimed at improving healthcare quality.

The competencies that received the highest level of evaluation in the studies included "medical knowledge" based on the ACGME classification, as well as "perform a clinical evaluation and establish a work plan" and "perform comprehensive treatment" based on the MINSA classification. Similar findings have been reported in other literature reviews, demonstrating that medical knowledge competencies are commonly assessed in various settings, such as among UK physicians [9] Physical Medicine and Rehabilitation residents [10], and during international surgery rotations [71].

The heightened attention given to medical knowledge competencies may stem from a concern for the acquisition of knowledge in comparison to other medical competencies. For instance, research conducted in Peru demonstrated that hospital resident physicians placed slightly greater emphasis on acquiring medical knowledge compared to other ACGME competencies [72]. Moreover, the prevalence and ease of access to assessment tools for these competencies may contribute to their prioritization over more complex clinical competencies, such as communication skills, shared decision-making, teaching, and others [73,74].

The competencies that received the least attention were "learning based on practice and improvement" and "systems-based learning" as per the ACGME classification. Similar findings in other reviews have identified "learning based on practice and improvement" as the least frequently assessed ACGME competency in studies [10,71]. It could be attributed to competence complexity and abstract nature, which presents challenges in its evaluation [75].

The competencies with the lowest level of evaluation based on the MINSA classification encompassed the areas related to generating new knowledge, applying technology and innovation, establishing cooperative relationships, and influencing and motivating individuals. The scarcity of research focusing on these proficiencies could be attributed to the belief that they are outlying from the practical aspects of medicine. Nevertheless, the ability to generate new knowledge, apply technology and innovation play a critical role in tackling health challenges in Peru [76]. Similarly, the capacity to establish cooperative relationships is fundamental for effective teamwork and collaborative decision-making within the medical context [77].

Regarding the assessment tools employed to evaluate competencies, most studies utilized surveys or exams. The advantage of these instruments lies in their ease of use, rapid administration, and cost-effectiveness. However, these instruments may not comprehensively assess medical competencies, as they rely on self-reports or specific knowledge [78]. Only four studies employed clinical scenarios like OSCE, Mini-CEX, or clinical simulation, for a competency evaluation. These methods are increasingly being adopted in various medical schools in Peru to assess clinical competencies [79,80]. Future studies should investigate the feasibility and accuracy of alternative instruments that may offer enhanced reliability, such as direct observation, simulated scenarios, or a combination of assessment tools [78]. Furthermore, there is a need to promote further research on the instruments’ development and adaptation to assess non-clinical competencies among Peruvian physicians.

Regarding funding, nearly one-third of the studies mentioned receiving funding from an institution. This finding is consistent with another study conducted at a Peruvian university, where one-sixth of scientific articles on medical education reported having funding [81]. However, medical education research in Peru and other Latin American countries still receives limited funding [82]. It could be attributed to medical education not being a priority within Peru’s biomedical research agenda [83], leading to reduced interest from funding agencies and medical researchers.

Among the 38 funded studies, six mentioned receiving funding from government entities and five from universities. Differently, a study analyzing 1822 articles found that governments and universities were the primary funding sources for medical education research published in high-impact scientific journals [84]. Exploring the role of these actors becomes important in promoting research on medical competencies.

It is recommended that medical education evaluative institutions in Peru, such as ASPEFAM (an institution assessing the theoretical knowledge of medical students in their final year of studies) and CONAREME (an institution conducting the entrance exam for resident physicians), incorporate the medical competencies addressed in this study into their assessments. Additionally, the evaluation of these competencies could be considered within undergraduate and postgraduate academic programs. This approach could enhance interest in this area, as researchers might seek to assess factors associated with the acquisition of such competencies and/or develop new measurement methods. Moreover, scientific output on medical competencies could be increased by employing alternative assessment methods, including those considering the perspective of patients. This could be achieved through the use of simple and reliable instruments such as surveys assessing communicative and interpersonal skills [85].

This study exclusively incorporated articles published in scientific journals, potentially neglecting pertinent unpublished studies. Furthermore, it concentrated on two standards, ACGME and MINSA, which are recognized as relevant to international and national contexts. Nevertheless, to our knowledge, this is the first study to map the scientific production of medical competencies in Peru. It allows the identification of existing gaps in the evaluation of medical competencies in Peru and further directs the strategies implemented to strengthen competency-based medical education, particularly in less evaluated competencies in practice. The use of the ACGME classification for medical competencies can be replicated in studies conducted in other countries. The obtained results reveal a scarcity of studies on certain medical competencies in Peru. This finding may reflect that the country is experiencing a delayed development in medical education compared to other countries, even within South America [86], a situation that might be similar to what occurs in countries with similar contexts.

## Conclusion

In conclusion, this scoping review characterized the published studies on professional competencies in physicians and final-year medical students in Peru. The studies primarily evaluated competencies related to medical knowledge (diagnosis, treatment), while behavioral competencies were less frequently assessed (ethics, professionalism, communication). Most studies were self-funded, conducted in Lima, and relied on questionnaires. It may be attributed to a scarcity of interest and resources dedicated to researching this topic. The results obtained reveal a scarcity of studies on specific medical competencies in Peru. This finding may reflect a similar situation in countries with comparable contexts.

## Supporting information

S1 Checklist Preferred Reporting Items for Systematic reviews and Meta-Analyses extension for Scoping Reviews (PRISMA-ScR) checklist. (DOCX)

S1 Table Operational definitions of each competence according to ACGME and MINSA. (DOCX)

S2 Table Search strategies used. (DOCX)

S3 Table Complete database. (XLSX)
