# Supplementary material for: Assessment of professional competencies of Peruvian physicians: A scoping review of published studies
Source: PLoS One. 2024 May 23;19(5):e0299465. doi: 10.1371/journal.pone.0299465 (PMC11115292; doi:10.1371/journal.pone.0299465)
Supplement: S1 Table — (DOCX) [file pone.0299465.s002.docx]

**Supplementary material 1: Operational definitions of each competence according to ACGME and MINSA.**

| **ACGME competencies** | **Definition** |
| --- | --- |
| 1) Patient care | It provides compassionate, appropriate, and effective patient care for health problem management and healthcare promotion. |
| 2) Medical Knowledge | It demonstrates biomedical, clinical, epidemiological, and social behavioral sciences knowledge. Further, its application to patient care. |
| 3) Interpersonal and communication skills | Demonstrates interpersonal and communication skills that lead to effective information sharing and collaboration with patients, their families, and healthcare professionals. |
| 4) Learning based on practice and improvement | Demonstrates the ability to investigate and evaluate patient care practice and assess and assimilate scientific evidence to improve patient care based on constant self-assessment and lifelong learning. |
| 5) Systems-based learning | Demonstrates awareness and responsiveness to the broader healthcare system and possesses the capability to efficiently utilize additional system assets in providing healthcare services. |
| 6) Professionalism | Demonstrates commitment to fulfilling professional responsibilities and adherence to ethical principles. |

| **Domains according to MINSA** | **Competences according to MINSA:** | **Definition (minimum conditions for the achievement of competence)** |
| --- | --- | --- |
| **Comprehensive health care for individuals throughout the life course, family, and community.** | 1) Perform clinical evaluation and establish a work plan | Develop the ability to conduct a comprehensive patient assessment, including medical history, physical examination, formulating diagnostic hypotheses, developing an approach plan, interpreting tests, documenting in medical records, and continuously re-evaluating. |
|  | 2) Provide comprehensive treatment for low/high-complexity healthcare problems. | Develop a rational and personalized treatment plan (prescribing medications, providing empathetic pain relief, informing patients and their families about the treatment, educating them on self-care, performing basic therapeutic procedures, CPR, and caring for and supporting individuals with terminal illnesses). |
|  | 3) Take actions for individuals' recovery with physical, mental, or social sequelae. | Guide the rehabilitation process through a patient-professional consensus plan, coordinate recovery actions, provide palliative care, and involve the family in the rehabilitation process. |
|  | 4) Promote changes in individual, collective, and environmental behavior. | Take care of one's health, educate for the adoption of positive practices, advocate with authorities to promote health care, organize community agent activities, and promote health policies. |
|  | 5) Carry out health interventions to reduce exposure, risks, and harm affecting individual and public health. | Implement interventions to address risk factors and minimize harm, participate in epidemiological and environmental health surveillance actions, prevent health risks from unnecessary procedures or treatments, and reduce the prevalence of health problems in apparently healthy individuals. |
| **Health system and care model** | 6) Practice the medical profession following the healthcare system of Peru | Recognize health as a right; promote actions that facilitate the reduction of barriers to access; engage in social transformation activities; utilize resources rationally; manage primary healthcare facilities; develop improvement plans; provide healthcare considering heterogeneity. |
| **Teaching and research** | 7) Participate in the education of students and the strengthening of healthcare human resources capabilities. | Planning, implementing, and evaluating teaching and learning activities, applying basic educational methods and strategies in daily practice. Ensure the safety and respect of healthcare users during training activities. |
|  | 8) Generate new knowledge that contributes to problem-solving in healthcare and decision-making. | Apply research approaches and methods, generating evidence that contributes to health improvement. Prioritize operational and applied research. Utilize professional practice as a research domain. Apply ethical principles in research. Write scientific documents for publications. |
| **Technology and innovation** | 9) Apply technology and scientifically grounded innovation. | Innovate in processes and resources; creatively adapt information and communication technologies (ICT); ethically and rationally use technologies with attention to new developments; proficiently handle ICTs, including telemedicine. |
| **Ethics and professionalism** | 10) Demonstrate commitment to the well-being and health of individuals and society. | Respect the rights of individuals. Make decisions and take actions considering the biopsychosocial model. Maintain a proper doctor-patient relationship. Respect and embrace cultural diversity. Practice the medical profession with honesty, integrity, commitment, humanity, empathy, and respect. Understand and uphold the Hippocratic Code. |
| **Communication** | 11) Establish professional relationships with individuals, families, and communities. | Apply the person-centered clinical method. Communicate effectively and assertively. Share information for health care. Interpret verbal and nonverbal communication. Adapt communication according to the listener. Utilize strategies for delivering bad news. |
| **Leadership** | 12) Influence and motivate individuals with respect and equity. | Contribute to achieving institutional performance standards. Promote a culture of respect and kindness. Engage social actors to address health issues. Manage conflicts within the healthcare team, and delegate tasks according to needs. Work collaboratively in a work team. |
| **Teamwork** | 13) Establish cooperative relationships, sharing knowledge and resources. | Recognize limitations, consult colleagues, and make appropriate referrals. Collaborate effectively within a multidisciplinary team. Share knowledge and resources, prioritizing the team's needs. |

MINSA: competencies in technical (in blue) and behavioral (in green) skills.
