# Supplementary material for: Assessment of professional competencies of Peruvian physicians: A scoping review of published studies
Source: PLoS One. 2024 May 23;19(5):e0299465. doi: 10.1371/journal.pone.0299465 (PMC11115292; doi:10.1371/journal.pone.0299465)
Supplement: S2 Table — (DOCX) [file pone.0299465.s003.docx]

**Supplementary material 2: Search strategies used**

| **Database** | **Search strategies** | **Number of results** |
| --- | --- | --- |
| **Pubmed** | (("Students, Medical"[Mesh] OR "Medical Student*"[Tiab]) AND (Intern*[Tiab] OR "Final Year"[Tiab] OR "Last Year"[Tiab] OR "Final-year"[Tiab] OR "Last-year"[Tiab])) OR ("General Practitioners"[Mesh] OR "General Practitioners*"[Tiab] OR "Physicians"[Mesh] OR doctor*[Tiab] OR "medical doctor*"[Tiab] OR "Internship and Residency"[Mesh] OR "Residency and Internship"[Tiab] OR Residenc*[Tiab] OR "Medical Internship*"[Tiab] OR "Internship"[Tiab] OR Specialist[Tiab] OR Physicians[Mesh] OR Physician*[Tiab]) AND ( Competenc*[Tiab] OR Abilit*[Tiab] OR Skill*[Tiab] OR "Knowledge"[Mesh] OR Knowledge*[Tiab] OR "Ethics, Research"[Mesh] OR "Research Ethic*"[Tiab] OR "Ethics, Clinical"[Mesh] OR "Clinical Ethic*"[Tiab] OR "Ethics, Professional"[Mesh] OR "Professional Ethic*"[Tiab] OR "Ethics, Medical"[Mesh] OR "Medical Ethic*"[Tiab] OR "Communication"[Mesh] OR "Health Communication"[Mesh] OR "Health Communication*"[Tiab] OR "Leadership"[Mesh] OR "Leadership"[Tiab] OR “technology”[tiab] OR “research*”[tiab] OR “teaching”[tiab] OR “innovation”[tiab] OR [tiab] OR "Social*"[Tiab] OR "Interpersonal*"[Tiab] OR "Communication*"[Tiab] OR Professionalism*[Tiab] OR "Professionalism"[Mesh] OR “Patient-physician relationship”[Tiab]) AND ("Peru"[Mesh] OR "Peru"[Tiab] OR peru*[Tiab])  Search date: February 05, 2023 | **238** |
| **Scopus** | (TITLE-ABS-KEY ("Medical Student" AND (Intern* OR "Final Year" OR "Last Year" OR "Final-year" OR "Last-year")) OR TITLE-ABS-KEY (physician* OR doctor* OR medic* OR "General Practitioner"* OR Residenc* OR "Internship" OR Specialist*)) AND (TITLE-ABS-KEY (Clinic* OR Procedur* OR Practic* OR Medic* OR Techn* OR "Healthcare Crew Resource Management" OR "Primary Healthcare" OR "Primary Care" OR Research* OR Ethic* OR Social* OR Interpersonal* OR Communication* OR "Systems-based Practice" OR "Patient care" OR Professionalism* OR “Leadership” OR “Teaching” OR “Innovation” OR “Patient-physician relationship”) AND TITLE-ABS-KEY (Competenc* OR Abilit* OR Skill* OR Knowledge*)) AND TITLE-ABS-KEY (Peru*)  Search date: March 01, 2023 | **93** |
| **Scielo** | ((internos) OR (médicos)) AND ((Perú) OR (peruanos)) AND ((competencias) OR (habilidades) OR (conocimientos))  Search date: March 01, 2023 | **32** |
| **Google Scholar** | **With boolean operators:** (“estudiante de medicina” OR intern* OR medico* OR resident* OR residenc*) AND (peru*) AND (competencia* OR habilidad* OR capacidad* OR aptitud* OR competencia* OR ética OR profesionalismo OR liderazgo OR “relación médico paciente” OR conocimiento*)  Search date: February 05, 2023 | **7780**  **(top 150 reviewed)** |
|  | **Without boolean operators:** médico estudiante de medicina competencias conocimiento Perú  Search date: February 05, 2023 | **31900**  **(top 150 reviewed)** |
|  | **Search for studies on the ENAM**  **Without boolean operators:** ENAM examen nacional de medicina Perú  Search date: March 25, 2023 | **19,100**  **(top 150 reviewed)** |
|  | **With boolean operators:** (“ENAM OR examen nacional de medicina”) + “Perú”  Search date: March 25, 2023 | **192**  **(top 150 reviewed)** |
